# Diagnostic Features and Potential Applications of PPG Signal in Healthcare: A Systematic Review

**DOI:** 10.3390/healthcare10030547

**Published:** 2022-03-16

**Authors:** Malak Abdullah Almarshad, Md Saiful Islam, Saad Al-Ahmadi, Ahmed S. BaHammam

**Affiliations:** 1 Computer Science Department, College of Computer and Information Sciences, King Saud University, Riyadh 11543, Saudi Arabia; saislam@ksu.edu.sa (M.S.I.); salahmadi@ksu.edu.sa (S.A.-A.); 2 Computer Science Department, College of Computer and Information Sciences, Al-Imam Mohammad Ibn Saud Islamic University, Riyadh 11432, Saudi Arabia; 3 The University Sleep Disorders Center, Department of Medicine, College of Medicine, King Saud University, Riyadh 11324, Saudi Arabia; ashammam2@gmail.com

**Keywords:** photoplethysmography, PPG, pulse oximeter, healthcare, wearable devices, cardiology, respiratory, neurology, diagnosis, monitoring, screening, fitness

## Abstract

Recent research indicates that Photoplethysmography (PPG) signals carry more information than oxygen saturation level (SpO2) and can be utilized for affordable, fast, and noninvasive healthcare applications. All these encourage the researchers to estimate its feasibility as an alternative to many expansive, time-wasting, and invasive methods. This systematic review discusses the current literature on diagnostic features of PPG signal and their applications that might present a potential venue to be adapted into many health and fitness aspects of human life. The research methodology is based on the Preferred Reporting Items for Systematic Reviews and Meta-Analysis (PRISMA) guidelines 2020. To this aim, papers from 1981 to date are reviewed and categorized in terms of the healthcare application domain. Along with consolidated research areas, recent topics that are growing in popularity are also discovered. We also highlight the potential impact of using PPG signals on an individual’s quality of life and public health. The state-of-the-art studies suggest that in the years to come PPG wearables will become pervasive in many fields of medical practices, and the main domains include cardiology, respiratory, neurology, and fitness. Main operation challenges, including performance and robustness obstacles, are identified.

## 1. Introduction

Photoplethysmography (PPG) measures the amount of light absorbed or reflected by human tissues. This optical waveform, sometimes called digital volume pulse (DVP) [1], is associated with the change in blood volume in the microvascular bed of tissue containing valuable information about the cardiovascular, respiratory, and nervous systems [2]. The importance of the PPG signal came from the fact that it can be easier to obtain with noninvasive and affordable sensors. PPG is commonly used in clinical practice to measure the oxygen saturation level (SpO2) in the blood and pulse rate as a vital sign of a patient. After reviewing the literature, we identified several other applications of this noninvasive method beyond pulse rate and SpO2. By processing PPG signals with different algorithms, researchers have acquired valuable information related to respiratory rate, blood pressure, ankle-brachial pressure, cardiovascular diseases, aging, neurological disorder, etc. 

**Figure 1:**
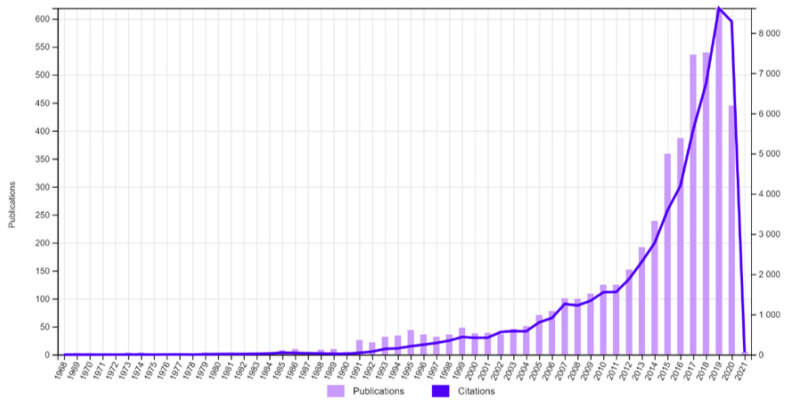
Web of Science publication and citation report, for the words “Photoplethysmography” OR “Photoplethysmogram”.

It is envisaged that the PPG signal can be clinically helpful to evaluate many physiological characteristics. It could play an important role in developing affordable and effective diagnostic, monitoring and screening tools in several areas of healthcare. Figure 1 shows the number of publications and citations of the words “Photoplethysmography” OR “Photoplethysmogram” from 1968 till now, from the Web of Science report. We relate the exponential growth around 2015 to the reveal of the first Apple watch.

Several reviews on the application of PPG signals have been carried out. Some of them focus on the specific medical use of PPG like pulse rate [3], blood pressure [4,5], atrial fibrillation [6,7], circulatory monitoring [8], nociception [9], or on the specific placement of PPG [10,11], others instead focused on reviewing the way that the signal has been analyzed [7,12,13,14,15,16] or the type of the sensor [17]. More than a decade ago, J. Allen [2] published an interesting review about the applications PPG in clinical physiological measurement. Kyriacou et al. [18] recently published a comprehensive book about Photoplethysmography theory and principles, providing a detailed description of PPG optical components and different signal analysis techniques. We will instead focus on PPG diagnostic aspects and healthcare applications. The main objective of our review is to investigate different types of diagnostic features and their potential applications on healthcare by reviewing the recent publications and highlighting the impact and challenges facing the deployment of PPG in healthcare.

In recent times, the PPG signal has been an important potential tool for diagnosis, monitoring, and screening of several cardiovascular, respiratory, and neurological diseases. Many of the analytical tools used in PPG studies have uncovered different diagnostic features useful for those applications. The robust analysis and classification of these signals is an important step towards developing affordable and convenient PPG-based tools in many applications. Towards this goal, a systematic review of the literature on measurable diagnostic features and usages in different clinical applications has been performed to address the following critical questions:

(1) What are the main diagnostic features of PPG signal related to different clinical applications?
(2) In which area can PPG signal have important impacts on future healthcare?

This systematic review of the literature on diagnostic features and potential applications of PPG signals in healthcare attempts to bridge this gap. The main objectives of this review are to classify the literature, highlighting trends, and criticalities concerning the use of photoplethysmography in healthcare. Besides, we tried to review the existence use of PPG in healthcare more broadly and systematically. We compiled all peer-reviewed published articles on the application of PPG signals and reviewed their diagnostic features and related clinical applications. By analyzing the overall trends and architectural comparisons made in individual studies, different PPG classification tasks were classified more effectively with specific architecture design choices. We hope that this review can serve as a starting point for future applications in architecture design of artificial intelligence and deep learning techniques for PPG classification.

The rest of the paper is organized as follows. In Section 2, we have revealed the methods in this systematic review. In Section 3, diagnostic features and current clinical usages are discussed. Section 4 listed the potential applications of PPG in health care in detail. Section 5 goes through the challenges. Finally, Section 6 is the conclusion.

## 2. Method of Systematic Review

A systematic literature search was done that involved searching and compiling available PPG literature. It was carried out to have enough background information and to answer our defined research questions, which would guide further research in the clinical use of PPG. The PRISMA 2020 guidelines for systematic reviews [19] were followed in order to achieve a valid formulation in this study. This review is divided into four main steps, as detailed below:

### 2.1. Search and Identification of Data Sources

A basic keyword-based search was made to gather as many publications as possible. We have looked up popular scientific databases and search engines, including Web of Science, Scopus, ProQuest, Elsevier, IEEEXplore, ScienceDirect, and ACM Digital Library using keywords “photoplethysmography” OR “photoplethysmogram” OR “PPG” OR “pulse oximeter” OR “photoplethysmographic” OR “oximetry”. 

This systematic review covers important PPG application literature with a more focus on recent research papers that have not been covered before in a review article. In addition, we also consider older research papers with significant contribution in the PPG research domain. Such extensive research returned almost 4000 scientific articles. 

### 2.2. Eligibility

For a more focused search, the following criteria were met:

- C1: Original research work published in peer-reviewed journals or conferences.
- C2: Publication date from 1981 (oldest PPG paper in Web of Science) to 2021.
- C3: More focus toward recent studies (from 2015 till now).
- C4: Important work with significant number of citations before 2015 that opened a new horizon in the field of PPG is considered.
- C5: If the keywords were not found in the title nor in the abstract, the paper was excluded.

After applying such criteria, the number of papers was reduced to around 500.

### 2.3. Selection Process

To ensure enough search coverage, we considered the important citations found in the original set of 500 papers. This process was done manually by screening each paper’s references list. 

**Figure 2:**
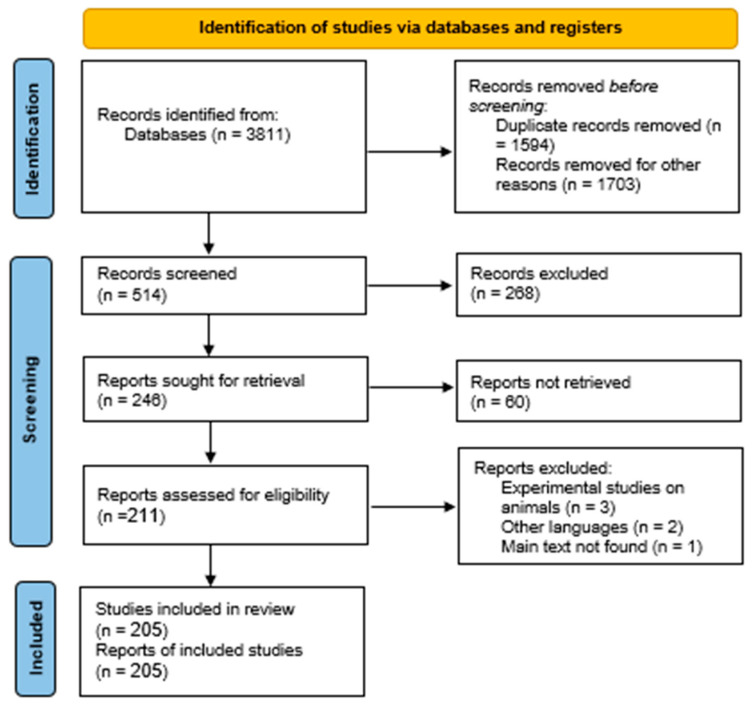
Systematic review flowchart according to PRISMA 2020.

All the abstracts were read and analyzed to finally decide the degree of relevance to our objectives. This was achieved considering four more criteria:

- C6: Only works that considered the use of PPG as one of their main contributions.
- C7: Papers must be original—duplicate or very similar work were excluded.
- C8: Only studies on the area of healthcare were included (other areas like industrial and education are not considered).
- C9: Studies on humans were considered only (other experimental studies on animals like pigs and cows are excluded). This leaves us with 205 articles shown in Figure 2.

### 2.4. Results

To investigate and analyze PPG healthcare applications, all papers were carefully read and classified based on their healthcare application domain. We tried to classify them as much as we could, based on the similarity of the papers, reaching roughly nine diagnostic feature and 12 potential applications, which finally falls under 4 main classes such as diagnosis, monitoring, screening, and others.

## 3. Diagnostic Features and Their Clinical Usages

**Figure 3:**
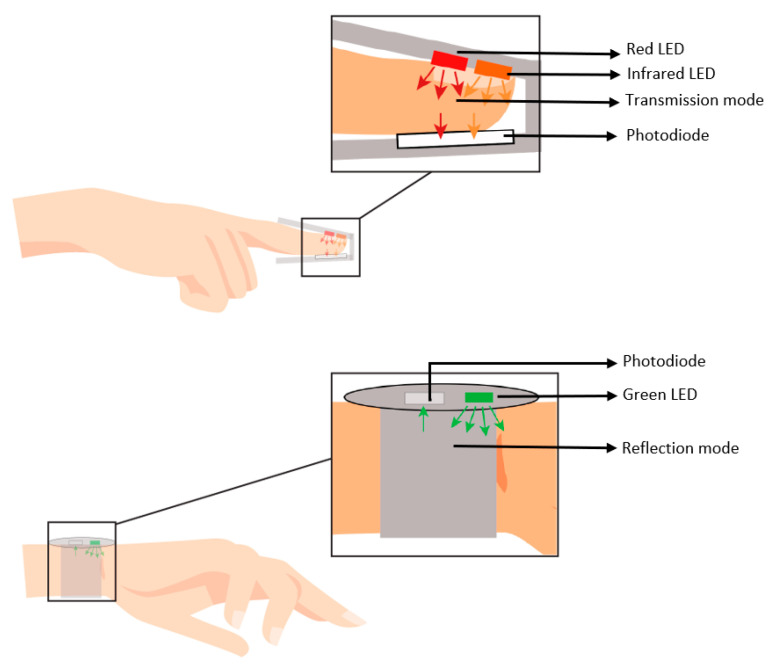
PPG transmissive mode vs. reflective mode.

The device used to capture photoplethysmograph is remarkably simple and inexpensive, and it consists of two components: a light-emitting diode (LED), which works as a light or laser source (sender), and a photodiode, which works as a light detector (receiver). Photoelectric plethysmography can be captured from fingers, toes, and nasal septum [20]. A PPG can be either transmission or reflection plethysmography (Figure 3).

Transmission plethysmography measures the LED light penetrating the body’s tissues, and it is usually suitable for the fingertip or earlobe. Reflection plethysmography, where the LED is next to the photodiode, continually records the scattering light from the tissue and usually is suitable for the forehead or chest [21]. 

Red and infrared light are commonly used in pulse oximeters [22] since they can penetrate human tissue deeper than green light [23]. The green light is often used in commercial wearable devices like smartwatches, because there is a buildup of experience from previous products to build on [23]. The PPG signal is clinically recorded using a pulse oximeter, usually with a clip attached to the index finger; other fingers can be used, even toes.

Imaging photoplethysmography (iPPG), also known as remote photoplethysmography (rPPG) or non-contact photoplethysmography, was introduced in 2000 by Verkruysse et al. [24,25]. The study shows that PPG signals can be remotely measured from a face image sequence taken by a regular video camera. Recently, a couple of mobile apps have put this technology within reach by taking advantage of the mobile camera to monitor pulse rate continuously. iPPG offers a solution to use a regular camera [26], a surveillance camera, or even a mobile camera to capture PPG from the face, forehead, chest, finger, or any exposed skin from different body parts. iPPG discovery opened up a new field of research and played an important role in remote patient monitoring experiments [14,27,28,29,30,31,32,33,34,35]. Deep learning hype is also brought to the field of iPPG [14,15].

**Figure 4:**
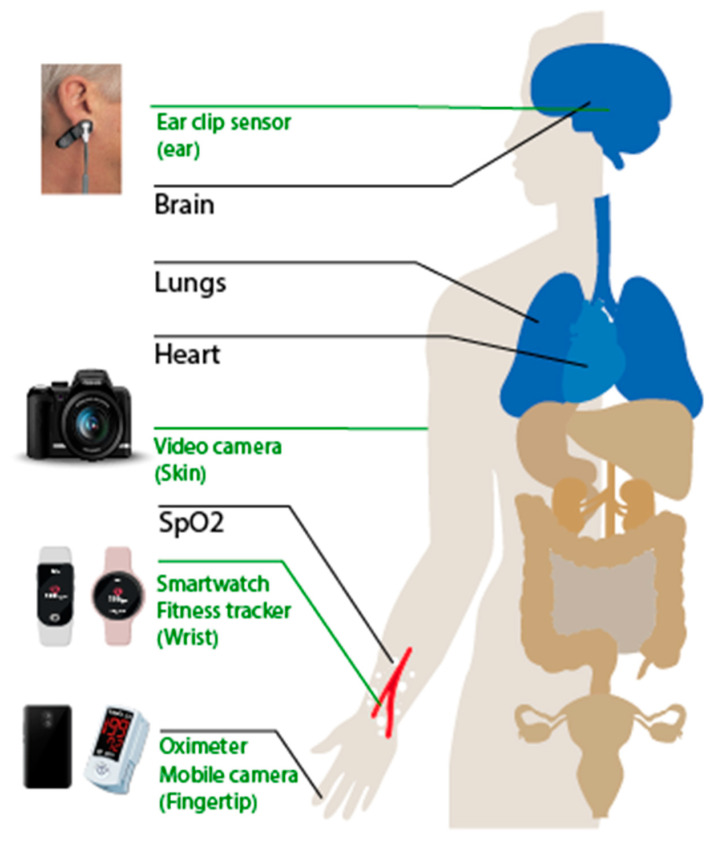
Different human organs affecting the PPG signal together with acquisition setups.

PPG signal is a combined result of different effects due to the activities of different organ systems. The light strength collected by the photodetector is affected by multiple factors, such as the blood volume, blood vessel wall movement, and the orientation of red blood cells [36]. There is a direct relationship between the PPG signal and human lungs and heart, and this relation is already used clinically. There is also an indirect relation between PPG and the human brain that is mainly used in observational studies [37,38]. The PPG signal might reflect other body organs’ status, but the question remains, is this relation reliable enough for diagnosing purposes? Figure 4 shows the important organs those have effects on the PPG signal. It also shows different PPG signal acquisition means.

**Figure 5:**
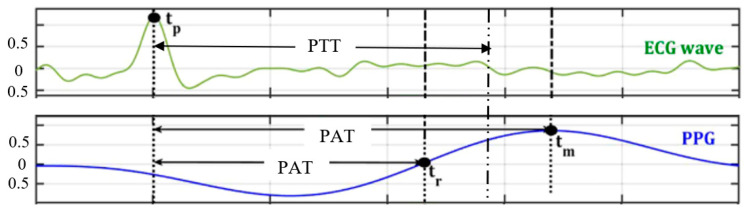
Synchronized ECG and PPG, and the PTT between the two signals. Note. Adapted from [43] Figure 2 “Blood Pressure Estimation Using On-body Continuous Wave Radar and Photoplethysmogram in Various Posture and Exercise Conditions”, by Pour Ebrahim, M., Heydari, F., Wu, T. et al., 2019, Sci Rep 9, 16346 (2019). (https://doi.org/10.1038/s41598-019-52710-8, accessed on 30 December 2021). CC BY 4.0.

There are a few main characteristics of photoplethysmography (PPG) waveforms, including amplitude (peak), height, area, width, maximum and minimum slope. These can be analyzed in time domain or frequency domain [1]. There are also other characteristics of PPG that were investigated widely in the literature, namely, pulse transit time (PTT), pulse wave velocity (PWV), and pulse wave amplitude (PWA). PTT is the time required for an arterial wave to propagate from two different arterial points. PTT can be measured using the time delay between ECG and finger photoplethysmography (PPG) waveforms (Figure 5). The relationship between PWV and PTT; that is, PWV = L/PTT, where L is the distance between the heart and some peripheral sites [39]. PWV can be calculated using PTT. It is noticeable that PTT is commonly used to estimate blood pressure [40], and PWV is commonly used to measure arterial stiffness [41]; both PTT and PWA are used during sleep studies [42].

In 1972, a Japanese study by Ozawa et al. [44] introduced the first and second derivatives of the PPG (SDPTG) [16] as a method to recognize the inflection points of the PPG wave more accurately. Since then, more researchers have relied on SDPTG as a prognostic tool for many diseases, especially cardiovascular diseases (CVDs) [45,46,47,48,49,50,51]. Several studies [45,47,49] show that SDPTG is significantly associated with CVD. Another study [50] also associated SDPTG with metabolic syndrome (MetS) and other cardiovascular risk indicators. While Terai et al. [52] pointed out that although SDPTG is not the most useful predictor of CVD in hypertensive patients, it might help in large population screening. Another usage of SDPTG was found on assessment of end-organ damage [53], and hemodynamic changes in dementia patients [54].

With the emerging integration of technology in every life aspect, wearable devices like smartwatches have become very popular due to the incorporation of PPG sensors. Many wearable-device manufacturers claimed that it could measure multiple health aspects like heartbeat, blood pressure, stress level during exercise. Devices with a PPG sensor could be worn on wrist, nasal [55], ankle, and chest [17]. Some researchers use PPG sensors attached to multiple body parts at the same time [56,57]. Others used unusual PPG sensor placement, like in bed mattress [58], body scale, or as a ring [59]. 

In the following subsections, we present the diagnostic features of PPG signal and their clinical application. In each subsection, the utilization of PPG in various health applications is discussed.

### 3.1. Pulse Rate and Its Variability 

Pulse rate (PR) and pulse rate variability (PRV) are the vital signs that are essential baseline evidence of patient health and useful for diagnosis, monitoring, and screening of different diseases. Two different approaches of measuring pulse rate are known: contact and non-contact PPG. In contact PPG [60,61,62], pulse rate is measured by placing a finger on the phone rear camera while, in non-contact [33,34,63], iPPG is extracted from the face, without the need for direct skin contact. In general, contact PPG-based apps showed better accuracy than non-contact PPG-based apps [64].

The use of iPPG obtained from a smartphone camera or from a regular camera to estimate PR is prevalent in the literature. PR was successfully extracted from the face using affordable cameras, and high degrees of agreement with the standard tools were achieved [26,65]. This was similarly true for iPPG from long-distance [66], and among the crowd [67].

Different methods of PR estimation were compared using iPPG from the DEAP dataset [31]. DeepPhys [68] is a deep convolutional network, proposed for heart and breathing rate measurement, tested against four datasets. In [69,70,71,72], a time-domain algorithm was proposed for real-time detection of the PR from wearable devices. Pulse rate variability can be detected from the SDPTG as well [48,49,50,51,73].

### 3.2. Blood Oxygen Saturation

In the early 1970s, Takuo Aoyagi, developed the first commercial oximetry, where PPG signal was obtained from the ear. In 1977, the Minolta Camera Company introduced the finger oximeter [44]. A normal oxygen reading of a pulse oximeter for healthy lungs usually ranges from 95% to 100%. Any abnormal reading is an alarm for several lung diseases, such as chronic obstructive pulmonary disease (COPD), pneumonia, and asthma.

Pulse oximeters were originally used in the operating room by anesthesiologists to observe unconscious patients. After that, the technology was approved for usage in the intensive care unit (ICU) and vital sign checking [44]. Nowadays, the PPG signal is practically used in clinical oximeters for oxygen saturation and pulse rate measurement in primary clinics and as a fitness indicator in smartwatches.

Measuring patient SpO2 using a finger is a standard practice in inpatient monitoring or outpatient checkup. When a patient has poor blood circulation in the fingers, an Ear Pulse Oximeter with an ear probe can be used on the earlobe. A couple of studies investigated PPG measurement behind the ear [74], and from the ear canal [75] compared to the finger oximeter; both indicated that blood SpO2 from the ear canal had a significantly faster response than finger oximeter. Digital video camera also can provide a rough screening tool for SpO2 [76,77,78,79].

### 3.3. Blood Pressure (BP)

Blood pressure (BP) evaluation is an essential measure in clinical practice. A patient’s BP is routinely obtained at every physical examination, including outpatient visits and inpatient monitoring. Many researchers evaluate the estimation of BP using PPG signal only [43,80,81,82,83,84,85,86,87,88,89], while others paired it with other types of signals, such as ECG [43,90,91] and BCG [40,92].

Many research on the usability of cuffless and continuous BP measurement has been done [40,43,80,82,83,84,85,86,87,88,89,90,91,92,93,94,95,96,97,98,99,100]. A smartwatch with two PPG sensors was used to estimate BP in real-time [81]. Wrist-worn bands [92,98], earlobe sensor [43] and even a special bathroom weighing scale [40] are used to estimate BP continuously. A couple of studies [97,100,101,102] monitor the BP iPPG from a camera; others used smartphone cameras [84,101]. 

BP is an identification of a wide variety of diseases, including hypertension, CVDs, and kidney failure [103]. Multiple papers [93,94,96] estimate BP using PPG in the Orthostatic hypotension test. Ishbulatov et al. [104] added CVD assessment to a similar study. Nuckowska et al. [95] studied slow breathing on BP measured by PPG. The vascular tone was continuously measured using PPG and was found to be higher in hypertension patients [105]. Some researchers use machine learning algorithms [106], such as deep convolutional autoencoder (DCAE) [87], Nearest Neighbor (NN), Support Vector Machines (SVM), Decision Trees (DT), Neural Networks [88,89,102,107,108], etc., to identify abnormal BP from PPG signal.

PPG-PB measurement was found to be comparable to the gold-standard sphygmomanometer reading; nevertheless, PPG is a more convenient method for long-term BP monitoring [81,98]. Although current technologies in measuring BP from PPG is not mature, it is anticipated that soon, accurate, continuous BP measurements will be within reach from mobile and wearable devices given their potentials [5].

### 3.4. Respiration Rate (RR)

The respiratory rate (RR), which is one of the vital signs, is the number of breaths in one minute. Pulse oximeter reading for normal resting heart for adults ranges from about 60 to 100 beats per minute, although some cases might be lower like athletes [22].

RR may differ if a person has a medical condition [109] and PPG waveform is also affected by respiration. Many methods were proposed to extract RR from PPG signal [70,71,110,111] and a real-time estimation of RR from PPG is presented in [70]. Extracted RR from finger PPG has better quality than extracted from ear PPG [110]. RR signal was also extracted from iPPG [112].

### 3.5. Arterial Stiffness and Aging

Aging is associated with major physiological and psychological changes in humans. Aortic pulse wave velocity (PWV) is used as the ideal measurement of arterial stiffness and PWV assessment can be acquired using PPG. Some studies try to investigate the effect of aging over PPG signal characteristics [113,114,115]. On the other hand, a couple of studies [45,56,57], examined the associations between aging and the SDPTG, to take advantage of the fact that SDPTG is used as an arterial stiffness indication. In fact, arterial stiffness tends to use SDPTG more than raw PPG. In 1998, Takazawa et al. [116] presented the second derivatives of the PPG as an index of vascular aging. 

Brillante et al. [117] consider PPG alone as a handy method of measuring arterial stiffness. They also conclude that arterial stiffness is correlated with age, race, cholesterol, blood pressure, and PR. Clarenbach et al. [118], Pilt et al. [119], and Wowern et al. [41], compared the advantages of the stiffness index (SI) derived from PPG and the augmentation index derived from arterial tonometry (AIx). SI and AIx are both used for the estimation of vascular stiffness degree. The three studies recommend the use of PPG over arterial tonometry. Since the SI method is fast, affordable, and operator-independent, it could implicate benefits in clinical use. 

PPG was used to predict AIx through a Deep Convolutional Neural Network (DCNN) [120]. To understand the evolution of arterial stiffness, Fung et al. [121] conducted a genome-wide association study for stiffness index (SI); 127,121 UK European participants were involved. The research recognized four loci significantly associated with SI. A system that uses eight PPG sites and 10 ECG sites was developed [56]. The main purpose of the system is to facilitate the assessment of arterial stiffness. A study by Arnold et al. [122], reported a relationship between inflammation, hemostasis, and arterial stiffness measured by PPG. The study emphasizes the ability of SI to indicate patients at higher risk of future CVD and increased mortality. Multi-site PPG system shows potential to assess cardiovascular risk linked to vascular aging [123]. Ohshita et al. [46], showed that post-challenge hyperglycemia is an independent predictor for arterial stiffness. 

### 3.6. Jugular Venous Pulse (JVP)

The jugular venous pulse (JVP) is useful to diagnose abnormalities in CVDs. The standard golden method to extract JVP is invasive central venous line catheterization. Several studies investigate noninvasive JVP measurement, using a contact PPG sensor placed on the neck [69] or a regular video camera [124,125,126]. Both of these approaches obtained remarkable results.

### 3.7. Ankle Brachial Index (ABI)

Ankle brachial index (ABI) is the ratio between BP in an artery of the ankle and the BP in an artery of the arm. Usually, it is done to check peripheral artery disease (PAD) [127]. However, some studies focused on its ability to indicate arterial stiffness [128,129]. The capability to predict ABI from PPG features was studied by Perpetuini et al. [129] and Cho [128].

### 3.8. Microcirculatory

The main usage of a pulse oximeter is monitoring blood perfusion in human tissue; precisely, it monitors the microcirculatory. A PPG sensor was attached to lower limbs to record perfusion increase [130]. iPPG was used to detect blood flow changes during thermal exposure to the skin [28]. It is also found that iPPG is also associated with cutaneous perfusion [32]. 

### 3.9. Autonomic Nervous System

An article by Aileni et al. [37] studied the correlations between biomedical signals (including PPG) that measure the electrical activity in the brain. Kawachi et al. [38] also proposed a new protocol to monitor the autonomic nervous system in patients with syncope that includes PPG.

Table 1 summarized PPG diagnostic features and their clinical application. Measurable diagnostic features based on different PPG waveforms such as contact-PPG, iPPG, and SDPTG are summarized in Table 2.

**Table 1:** Measurable diagnostic features from different organ systems and their clinical applications.

Measurable Features	Clinical Applications		
	Diagnosis	Monitoring	Screening
**Pulse rate and its variability**	-	PR [33,34,61,63,64,65,68] PRV and CSC [62] CVD [66] Vital sign [26,67]	PR [31,60]
**Blood oxygen saturation**	-	SpO2 [74,75,79]	SpO2 [76,77,78]
**Blood pressure**	Hypertension [5,99] Orthostatic hypotension [93,94,96] CVD [104,105]	CVD [91] BP [40,43,80,81,82,83,84,85,86,87,88,89,90,91,92,93,94,95,96,97,98,99,100,102,106,107,108]	-
**Respiration rate**	-	RR [70,71,110]	-
**Arterial stiffness**	Aging [45,57,113,114,115] PWV [117] SI [41,118] CVD [56,122] Vascular aging [116]	SI [46,123] Cardiovascular risk [123]	SI [119,121] AIX [120]
**JVP**	-	JVP [69,124,125,126]	-
**ABI**	CVD [119,120]	-	-
**Microcirculatory**	-	Perfusion change [28,32,130]	-
**Automatic Nervous system**	Electrical activity in the brain [37]	Syncope [38]	-

**Table 2:** Measurable diagnostic features based on different PPG waveforms.

Measurable Features	Signal Type		
	PPG	iPPG	SDPTG
**Pulse rate and its variability**	[50,64,69,70,71,72]	[26,31,33,34,60,61,62,63,65,66,68]	[48,49,50,51,73]
**Blood oxygen saturation**	[74,75]	[76,77,78,79]	-
**Blood pressure**	[40,43,80,81,82,83,84,85,86,87,88,89,90,91,92,93,94,95,96,97,98,99,100,102,106,107,108]	[84,97,100,101,102]	-
**Respiration rate**	[70,71,110]	[68,112]	[111]
**Arterial stiffness**	[41,113,114,115,117,118,119,120,121,123]	[35,126]	[45,46,56,57,116]
**JVP**	[69]	[124,126]	-
ABI	[128,129]	-	-
**Microcirculatory**	[130]	[28,32]	[53,54]
**Automatic Nervous system**	[37,38]	-	-

## 4. Potential Applications of PPG Signal and Impacts on Healthcare

Today PPG found its applications in several sectors of healthcare, from vital sign estimation like pulse rate, respiratory rate, and blood pressure to more complicated diagnosis areas like sleep staging and arterial steal syndrome. In 2015, the Apple Watch (AW) with embedded PPG was released, and since then, it has become the leading personalized wearable device with a touch of fashion. In 2018, the United States Food and Drug Administration (FDA), identified AW as “PPG-AFib analysis software for over-the-counter use” [131]. 

It could be noted that some medical applications are directed into the use of PPG signals more than others, like CVDs and sleep disorders. In the following subsections, we discuss the potential clinical applications of photoplethysmography in diagnosis, monitoring, screening and fitness.

### 4.1. Diagnosis 

Adapting wearable devices that use PPG sensors contributes to early diagnosis and prevents further health complications. In addition, it provides a continuous and convenient observation. PPG wearables show great potential to be used in CVDs risk estimation [118], prognostic of suspected sleep apnea [132,133], and the clinical diagnosis of hypertension [104], diabetes and psychiatric conditions. A combination of PPG and acoustic Doppler analysis provides a reliable method for arterial steal syndrome (ASS) and arteriovenous fistulas stenosis detection in patients receiving hemodialysis [134].

#### 4.1.1. Cardiovascular Diseases (CVDs)

According to World Health Organization (WHO), cardiovascular diseases (CVDs), are the leading cause of death globally. It includes a group of heart and blood vessels disorders, such as: heart attack, stroke, and heart failure [135]. Cardiology is one of the primary application areas of PPG signal for diagnosis, monitoring, and screening of cardiovascular diseases (CVDs). Noninvasive and continuous CVD risk assessment and management have gained a lot of attention recently, especially after news about commercial smartwatches have been life-saving for multiple unaware CVD patients.

PPG signal and SDPTG have been utilized to diagnose several CVDs. It was found that there is a noticeable association between the SDPTG and cardiovascular mortality [51]. The authors of [136] classify the output of diagnosis of CVDs in PPG signals using a fuzzy model. A PPG device prototype was designed to identify heart failure (HF) [137]. PPG was used to monitor obstructive sleep apnea in subjects with HF [138]. It was used to remotely monitor jugular venous on HF patients [139]. It has also been studied and investigated to measure other cardiac disorders, e.g., venous occlusion [140] and obstructive hypertrophic cardiomyopathy [141], and to predict cardiac indexes, e.g., cardiac output [142] and arterial compliance [143]. The stiffness index derived from PPG is simple and possibly yields an advantage in CVDs risk estimation [118].

However, there is an evident need for further studies as well as tests before any such employment could be made available. In addition, future works aiming to do other analysis technics are needed, especially with deep learning, for a better classification of CVDs.

#### 4.1.2. Sleep Disorders

Polysomnography (PSG), or sleep study, is an overnight test where the patient is traditionally admitted to the hospital. It is used for diagnosing sleep disorders, including obstructive sleep apnea syndrome (OSAS). PSG needs a lot of resources that place a large burden on patients and healthcare providers at the same time [144]. 

iPPG enables continuous and convenient monitoring of pulse rate and oxygen saturation during sleep [145]. Using PPG to diagnose sleep disorders has been widely investigated [42,132,138,146,147]. PPG is used to assess a patient’s blood pressure [42] or cardiorespiratory status while sleeping [42,146]. Korkalainen et al. developed an accurate deep learning model for recognizing sleep stages based on PPG [132].

The potential of having a portable and convenient method to diagnose sleep disorders is motivating; it complements the standard Polysomnography (PSG) diagnostic techniques by allowing unobtrusive sleep and respiratory monitoring.

#### 4.1.3. Diabetes 

Studies investigating non-invasive blood glucose monitoring are still in the early stage, and the relation between blood glucose and PPG obtained from a pulse oximeter is ambiguous. A study in 2012 [148] reported a false reading of pulse oximeter when used in a diabetic patient under oxygen therapy. However, some researchers use diabetes patients as research subjects [149,150,151,152,153,154]. Others use machine learning models, like support vector machine [155] and logistic regression [156], to differentiate between PPG signals for diabetic patients and non-diabetic patients. With the aid of machine learning techniques, PPG shows advances in measuring glucose levels [157,158]. DNN is used to build a biomarker of diabetes from PPG signals collected using a smartphone app from 53,870 participants [159]. PPG readings from the diabetic neuropathic group were different from those from the control group [149,150,151,160]. 

#### 4.1.4. Psychiatry

According to the American Psychiatric Association [161], psychiatry is “the branch of medicine focused on the diagnosis, treatment, and prevention of mental, emotional and behavioral disorders”. Human emotions and expressions explanation is confusing and interesting even for other humans. Humans’ ability to lie or pretend gives them the power to hide their real emotions, but physiological signs cannot lie. In fact, PPG signal has emerged as a new tool to detect human emotions, anxiety, and mental stress.

Human reactions towards video clips have been studied using biosignals, including PPG in [162,163,164]. Lee et al. [162] studied drivers’ anxiety in different situations, while Tanaka et al. [165] evaluated patient anxiety before an operation by examining vascular tone taken from finger PPG. Recognizing drivers’ emotions toward a video clip using PPG is possible [162], but it is challenging to classify their physiological response using PPG because of the motion artifact effect [166]. A study [29] used iPPG to confirm that physiological changes are related to spontaneous expressions. Perpetuini et al. [167] suggested that PPG can be used in emotions recognition and estimate the state anxiety, in the field of human–machine interaction. Fernández et al. [163] differentiate between subjects’ responses based on the age group. KEmoCon [168] is a dataset that used PPG along with other physiological signals to record the emotions of 32 participants in the context of social interactions.

In order to assess mental stress from PPG, Charlton et al. [169] identify features of PPG that can indicate mental stress. A PPG earlobe sensor was used to monitor PRV induced by mental stress [170]. A person’s stress state was classified using convolution neural networks [171]. Cognitive load increases with the challenging level of a game; PPG signals can measure cognitive load during video game playing [172]. iPPG can predict PRV remotely under cognitive stress [173,174,175] and under mental arithmetic test [176,177]. iPPG system can be used to measure peripheral hemodynamics during psychological stress without contact [27].

Raheel et al. [164], conclude that PPG classifies human emotions better than electroencephalography (EEG) and galvanic skin response (GSR). However, the research in this area is yet nascent or immature; researchers do not know what psychiatry issues may emerge from PPG data and avoid hypothesizing particular relationships between variables. More efforts toward specific subtopics can reduce the fuzziness of the literature.

### 4.2. Monitoring

Patients admitted in the hospitals consume the most part of healthcare resources, especially critical care patients, where they need continuous monitoring, that’s required complex and expensive devices, and around-the-clock nursing staff and physicians. PPG-based continuous monitoring offers affordable, accessible, convenient, and reliable long-term monitoring systems. PPG is also used to monitor patients in the intensive care unit (ICU) and the Neonatal Intensive Care Unit (NICU) [134,178,179].

#### 4.2.1. Outpatient Care (OPD)

Outpatient services are usually done in a medical center. This includes tests, minor procedures, scenes, and doctor consultations. Wearable devices such as smartwatches and fitness bands could provide continuous patient monitoring without the need for admission [45], particularly for chronic disease that requires ongoing medical attention, such as heart disease [180,181], and peripheral neuropathy [149]. Terminal illness care can be provided at the convenience of home with the evolution of PPG wearables [54,58].

Continuous health monitoring for the management of chronic diseases through wearable PPG devices is more reasonable than iPPG solutions. On the other hand, iPPG solutions through cameras are more suitable for population screening.

#### 4.2.2. Pediatrics

Pediatrics is a branch of medicine that deals with physical, mental, and social health of children from birth to maturity. Children differ from adults in many health measurements aspects [182]. Different vital signs induced from the iPPG signal of children are strong enough to be measured. iPPG offers contactless monitoring which is important in pediatrics especially monitoring NICU patients. 

For young children, the measurement of the electrical activity of the heart is possible by using PPG-based system [183] as an alternative of ECG [184]. Another study in [185] shows the feasibility of using PPG to estimate PRV at home for pediatric oncology. A couple of papers indicates the possibility to measure BP using PPG in toddlers [186], and obese children [181]. PTT derived from PPG can be used as a noninvasive measure of cardiovascular risk, especially in infants and toddlers [187]. 

Monitoring NICU patients’ cardiovascular [188,189], respiratory [190], and circulatory parameters [189,191] is possible through PPG oximeter. iPPG also can aid in monitoring SpO2, PR, and RR status from a video camera [78,191]. Pulse transit time of PPG may aid in evaluating Patent Ductus Arteriosus (PDA) status in preterm infants [192]. Chung et al. [193], designed a small PPG-based, noninvasive, and relatively small adhesive bandage that can be used in NICU or pediatric intensive-care units PICU for vital signs continuous monitoring.

Challenges like low light level and child motion prevented successful and continuous measurement from time to time. Better hardware and improved algorithms are required to increase robustness.

#### 4.2.3. Surgery

A pulse oximeter is one of the essential instruments that are attached to an anesthetic patient during surgery. PTT is an effective way to evaluate the level of analgesia for patients performing hand surgery [194]. A study investigated the characteristics of low-frequency (LF) pulse rate variability (PRV) and PPG waveform change during cardiac surgery and revealed that the synchronization between PPG and PRV probably came from neurogenic nature [195]. A nasal PPG instrument was developed to evaluate analgesic levels during surgery for patients under general anesthesia [55]. Chen et al. [179] found an association between PPG and major hemorrhage in trauma patients during medical helicopter transport.

iPPG obtained from the chest can be used after major surgery to assess the differences in cutaneous perfusion [30]. It is also used to assess blood perfusion in the brain cortex during open brain surgery [196]. PulseCam [197] is a sensitive camera that can capture blood perfusion conveniently and affordably. 

#### 4.2.4. Remote Monitoring during COVID-19 

While the world is still suffering from the pandemic of COVID-19, finding a convenient, cheap solution would save healthcare providers a lot of effort and funds. SpO2 reading is one of the first signs to be checked in ER to decide COVID-19 severity [198]. To assess patient condition; healthcare workers should evaluate pulse-oximetry for individuals presenting with respiratory symptoms, fatigue, or malaise. iPPG systems and PPG wearable sensors provide a convenient remote way to monitor patient vital signs, especially COVID-19 patients. It also could facilitate keeping patients away from medical staff. PPG wearables can measure a patient’s PR, BP, SpO2, and respiratory rate [75,199,200]. 

Recently FDA published several guidelines and an enforcement policy [201] for noninvasive remote monitoring devices manufacturer regarding COVID-19. This highlights the important role of wearable devices such as smartwatches in monitoring patient health in such pandemics. Apple website [202] announced detailed instructions for patients and health care providers to emphasize the usefulness of the watch in telemedicine visits during COVID-19. There is also a need to develop algorithms that can indicate the possibility of infection in the early stages of COVID-19. Additionally, the interesting idea is to estimate the quarantine duration for each individual using wearable devices [199].

More studies are needed to determine the feasibility and validity of iPPG systems for mass screening against COVID-19. Although these systems may be used for initial individuals’ temperature evaluation, in high throughput places like airports, shopping malls, and sporting events, their validity, when used for multiple people simultaneously, needs further investigation.

#### 4.2.5. Neurology

Neurology is the branch of medicine concerned with the diagnosis and treatment of nervous system diseases that affect the brain, the spinal cord, and the nerves [203]. It includes pain, headache, sleep, epilepsy, etc. [204]. Wearable PPG sensors can be promising to monitor Alzheimer’s patients [58], and multiple sclerosis patients [205]. Finger SDPTG, which provides hemodynamic changes, was studied in Alzheimer’s and Binswanger’s patients [54]. 

The visual analog scale or the numeric rating scale is generally used in clinical practice to assess pain severity, but these scales cannot be used for unconscious patients or those with dementia [206]. Several publications have appeared trying to measure pain using PPG and putting patients under different stimuli, e.g., electrocutaneous [206], heat [207], stimuli images [208], and pain provoked in a social experiment [209].

#### 4.2.6. Dialysis

Wieringa [210] suggested a wide adoption of smart sensors for patients on dialysis (including PPG), as they need continuous monitoring to optimize treatment efficacy and patient care. Arterial steal syndrome is one of the dialysis complications. PPG can be used for arterial steal detection [134].

### 4.3. Screening and Fitness

PPG signals collected from mobile phone applications can be very beneficial to build standard health indices and measurements nationwide. Considering the affordability and availability of digital cameras everywhere, iPPG has tremendous potential for the future of mass screening. The Huawei Heart Study provided an excellent example of the benefits of PPG to perform general population screening [211]. 

#### 4.3.1. Atrial Fibrillation (AFib)

Atrial fibrillation (AFib) is defined as an abnormal and often rapid heartbeat. It has clear connections with other CVDs, including HF, diabetes mellitus, and hypertension [212]. Commonly used methods to detect AFib are instant ECG examination and 24 h ECG Holter observation. However, due to its clinical limitation, the ECG method might miss some AFib episodes [178]. On the other hand, on the standard clinical monitor, PPG is an outstanding indicator of cardiac arrhythmia in general. PPG is highly sensitive to any irregularity of the pulse [213].

Detecting AFib using wearable devices is a feasible and reliable approach during inpatient physical activity [214] or free-living conditions [180,215,216]. However, the accuracy is degraded based on the intensity of the movement [162]. An interesting AFib risk score assessment is developed in Huawei Heart Study on 644,124 individuals [211]. The result of the study could ease future population screening and prevention against AFib. DeepBeat [216] is a multi-task deep learning model to estimate signal quality and detect arrhythmia episodes simultaneously in wearable PPG. In 2017, Tang et al. [178] proposed a novel clinical study to investigate the effectiveness of PPG signals in identifying AFib. The study simultaneously recorded ECG and PPG signals in patients admitted to the ICU with a stroke. Taking into consideration the need for annotated, public PPG databases with an abnormality, Sološenkoa et al. [217], used ECG databases with AFib episodes to generate artificial PPG signals.

Although the above research indicated that PPG could correctly detect AFib most of the time, this sort of application still needs to be tested in a larger population. There is a need to prove that PPG can differentiate between AFib and other kinds of arrhythmias. However, if future trials prove successful, it could be a cost-effective, convenient way to screen people early for this common problem.

#### 4.3.2. Fitness

Smartwatches and fitness trackers are usually advertised as personal assistants and health monitor devices that can count your steps, calories and measure pulse rate. These PPG-based technologies have the ability to enhance the quality of individuals’ life. The elevation of personal lifestyle and fitness level have a direct impact on the public health level.

Fitness smartwatch is the most common application for PPG usage in daily life. It can measure SpO2 and PR continuously and accurately. PPG waveform changes while exercising, which might be useful for measuring cardio stress [218], and cardiac output [142]. ‘Heartbeats’ is a system designed to produce different kinds of music based on synchronized PPG, based on the claim that music can enhance athletic performance [219].

### 4.4. Others

There are other medical applications of PPG waveform that have not gained much attention, like fertility monitoring [220], end-organ damage [53], wound healing prediction [221], and hematologic disorder [222].

**Figure 6:**
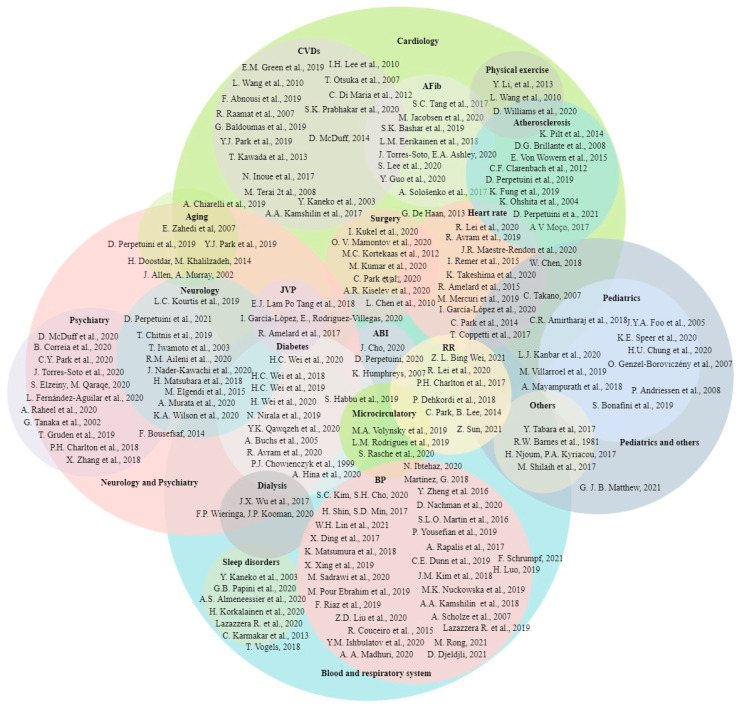
Venn diagram representing an overview of the PPG healthcare applications [5,26,27,28,29,30,31,32,33,34,35,37,38,40,41,42,43,45,46,47,48,49,50,51,52,53,54,55,56,57,58,60,61,62,63,64,65,66,67,68,69,70,71,72,73,74,75,76,77,78,79,80,81,82,83,84,85,86,87,88,89,90,91,92,93,94,95,96,97,98,99,100,101,102,103,104,105,106,107,108,109,110,111,112,113,114,115,116,117,118,119,120,121,122,123,124,125,126,127,128,129,130,132,134,136,143,145,146,147,148,149,150,151,152,153,154,155,156,157,158,159,160,162,163,164,165,166,167,168,169,170,171,172,173,174,175,176,177,178,179,180,181,183,184,185,186,187,188,189,190,191,192,193,194,195,196,197,198,199,205,206,207,208,209,210,211,214,215,216,217,218,219,220,221,222].

Table 3 summarizes the main application areas of PPG together with different types of signal such as contact-PPG, iPPG, and SDPTG. Table 4 lists specific applications of different PPG in employment areas. Finally, Table 5 presents public datasets of PPG signal used in the literature. The Venn diagram in Figure 6 illustrates literature classification based on healthcare application domains.

**Table 3:** Different PPG waveforms and their application based on health domain.

Application		Signal Type		
		PPG	iPPG	SDPTG
**Diagnosis**	CVDs	[136,137,138,139,141,142,143]	[140]	[49,50,51,52]
	Sleep disorders	[42,132,138,146,147]	[145].	-
	Diabetes	[148,149,150,151,152,153,154,155,156,157,158,160]	-	-
	Psychiatry	[162,163,164,165,167,169,170,171,172]	[27,29,173,174,175,176,177]	-
**Monitoring**	OPD	[58,149,180,181]	-	[54]
	Pediatrics	[181,184,186,187,188,189,190,192,193]	[78,191]	-
	Surgery	[55,179,195]	[30,196,197]	-
	COVID-19	[75,199,200]	-	-
	Neuro	[58,205,206,207,208,209]	[196]	[54]
	Dialysis	[134,210]	[210]	
**Screening**	AFib	[180,214,215,216,219]	-	-
	Fitness	[142,218,219]	-	-
**Others**		[220,221,222]	-	[53]

**Table 4:** Specific applications of PPG signal in healthcare in each domain.

Application		Usages (Related Disease)
**Diagnosis**	**CVDs**	Cardiac output [136,142] HF [137] OSA [138] JVP [139] Venous occlusion [140] Obstructive hypertrophic cardiomyopathy [141] Arterial compliance [143]
	**Sleep Disorders**	BP and PR [138] BP [42] CVD [42,146] Sleep staging [132] Apnea [133,138,147]
	**Diabetes**	Diabetes [155,156,157,159] Glucose level [157,158] Diabetic neuropathy [149,150,151,160]
	**Phychiatry**	Reactions [162,163,164]. Anxiety [162,165,167] Vascular tone [165] Emotions [162,164,167]. Physiological response [29,163,166] Age [163] stress [27,169,170,171] Cognitive load [172,174,175,176,177]
**Monitoring**	**OPD**	CVDs [180,181] Peripheral neuropathy [149] Dementia [54,58]
	**Pediatrics**	PRV [183,185] BP [181,186] CVD [187,188,189] RR [190,191] PDA [192] Vital signs [193]
	**Surgery**	Anesthesia [55,194] PRV [195] Blood perfusion [30,179,196,197]
	**COVID-19**	PR, BP, SpO2, & RR [75,199,200]
	**Neuro**	Dementia [54,58] Multiple sclerosis [205] Pain [206,207,208,209]
	**Dialysis**	treatment efficacy [210] Arterial steal [134]
**Screening**	**AFib**	detect episodes [162,178,180,211,214,215,216]
	**Fitness**	Exercising [142,218,219].
	**Others**	Fertility monitoring [220] End-organ damage [53] Wound healing prediction [221] Hematologic disorder [222]

**Table 5:** Public datasets of PPG signal used in the literature.

Area	Databases
**CVDs**	CapnoBase [223] used in [136] PPG-DaLia [224] used in [72]
**Psychiatry**	K-EmoCon used in [168] for continuous emotion recognition
**Neuro**	International Affective Picture System (IAPS) [225] used in [208] for image stimuli.
**AFib**	2015 IEEE Signal Processing Cup [226] used in [216] for AFib episodes detection MIMIC and the University of Queensland Vital Signs Dataset [227] used in [217] for AFib modeling

## 5. Challenges

As we have shown before, the use of the PPG waveform could be useful for many healthcare applications. However, it is not adopted yet in actual practice in most of the domains due to several limitations and challenges.

1. Approvals and authorities: Although wearable devices have gained popularity among a wide range of users to track their health and fitness, they need to seek health authority’s approval to provide better, trusted solutions and avoid future legal issues.
2. Pulse oximeter clinical limitations: As the most used PPG device, the pulse oximeter has a major limitation. The pulse oximeter is used to measure the SpO2 of the hemoglobin in arterial blood, but it does not necessarily reflect how well the patient is ventilated. Even when the SpO2 reading is normal, the pulse oximeter cannot distinguish between blood saturated in oxygen or in carbon monoxide, which explains the false positive reading obtained right away after smoking [22]. Another medical limitation that clinicians must be aware of is the false reading for low peripheral vascular perfusion patients.
3. PPG alone is not always enough: Usually, investigating the efficacy of PPG signal is compared or paired with other biological signals or diagnosing methods that are commonly used in practice. For example, ECG is the most common signal that is usually combined with PPG; for sleep disorders, it is combined with PSG, or with Electroencephalography (EEG) for neurology research. Therefore, after all, PPG alone might not be enough, especially if we need a very accurate measurement.
4. Abstract representation: Clinical oximeter should be able to show more detailed information about the PPG signal to the practicing clinicians [213]. Sometimes irregular or anomaly readings of an oximeter could be found without a convincing explanation.
5. A hybrid field: Further research effort and a much greater focus on collaboration between healthcare and engineering is needed to facilitate the adoption of wearable devices in practice. Experts from both specialties are needed to reach an outstanding outcome.
6. Quality of the acquired PPG wave: Another challenge facing the adoption of PPG-based devices is that PPG signal quality might be reduced due to many artifacts. Motion artifacts can interfere with the proper acquisition of reliable PPG signals. Several approaches in the literature deal with the corruption of the signal using signal processing algorithms.
7. The emergence of machine learning: Although multiple studies showed great performance by applying deep learning to PPG signal, that area could face a couple of challenges, including the required computation power and data annotation, besides dealing with other wearable device limitations such as storage and battery.
8. Shortage of data in medical applications: Although there are a couple of medical datasets that contain a PPG signal filed, three of them are drawn from the Medical Information Mart for Intensive Care (MIMIC) database [228] with an alteration. There is a lack of a PPG dataset with a large sample space in many healthcare topics.
9. Patient privacy and security: Wearable devices such as smartwatches raise multiple security issues; before developing a remote medical system, multiple issues shall be kept in mind, like insecure wireless connection, lack of encryption, and authentication.

## 6. Conclusions

PPG signal lately gained immense attention due to the availability of wearable sensors and oximeters. This survey has revealed the diagnostic features of PPG signals besides their potential clinical usage in healthcare. We have reviewed the potential impact of PPG signals for diagnosing, monitoring, screening and fitness of inpatient and outpatient. To aid future research, we have identified potential challenges that future adoption might face. Although the investigation of PPG-based methods in diagnosis and monitoring is not nascent, more collaboration between medical practitioners, engineers, and wearable sensors manufacturers could give a jumpstart.

## Data Availability

All data generated or analyzed during this study are included in this review.
